# Preparation and Evaluation of Amino Acid Conjugates of Celecoxib as Prodrugs to Improve the Pharmacokinetic and Therapeutic Properties of Celecoxib

**DOI:** 10.3390/pharmaceutics12111043

**Published:** 2020-10-30

**Authors:** Yonghyun Lee, Jungyun Kim, Wooseong Kim, In-Soo Yoon, Yunjin Jung

**Affiliations:** 1 College of Pharmacy, Pusan National University, Busan 46241, Korea; sthgood123@naver.com (J.K.); b04420@nate.com (W.K.); insoo.yoon@pusan.ac.kr (I.-S.Y.); 2 Department of Pharmacy, College of Pharmacy, Ewha Womans University, Seoul 03760, Korea; 3 Graduate School of Pharmacy, Ewha Womans University, Seoul 03760, Korea

**Keywords:** celecoxib, prodrug, bioavailability, PK linearity, extended absorption, carrageenan-induced rat paw edema model

## Abstract

Although celecoxib is quite effective in the management of inflammation-related diseases, especially arthritis, its use is limited by concerns including low bioavailability (BA), non-linear pharmacokinetic (PK) profile, and peak concentration-related toxicity. To overcome these issues, we designed and prepared hydrophilic celecoxib prodrugs, namely *N*-glycyl-aspart-1yl celecoxib (*N*-GA1C), glutam-1-yl celecoxib (G1C), and aspart-1yl celecoxib (A1C), for the sustained release of celecoxib in the intestine with limited systemic absorption. The celecoxib derivatives were converted to celecoxib in the intestinal contents. The conversion rates were in order of N-GA1C > G1C > A1C. Oral administration of the celecoxib derivatives (oral celecoxib derivatives) sustained the plasma concentration of celecoxib for 24 h, improving the BA and linearity of the PK profile of celecoxib. The peak concentrations (C_max_) of celecoxib after oral celecoxib derivatives were lower than that after oral celecoxib. In a carrageenan-induced rat paw edema model, oral N-GA1C exhibited greater anti-inflammatory activity for a longer duration compared with oral celecoxib. The order of efficacy of the celecoxib derivatives was N-GA1C > G1C > A1C. Taken together, the prodrug approach is a feasible strategy to improve the PK and therapeutic properties of celecoxib, and among the celecoxib derivatives, N-GA1C may be the most promising prodrug of celecoxib.

## 1. Introduction

Celecoxib, a selective COX-2 inhibitor, is one of the most popular anti-inflammatory drugs for the treatment of chronic inflammatory diseases such as arthritis [1,2]. Celecoxib is a Biopharmaceutics Classification System (BCS) class II drug with low solubility and high permeability [3,4]. Despite the extensive clinical use of celecoxib, some negative pharmacokinetic (PK) properties, such as (1) low bioavailability (BA) [3,4,5], (2) non-linear pharmacokinetic profiles [6], and (3) peak blood concentration-related cardiovascular toxicity [2], have hindered its broad clinical usage.

Generally, a prodrug, pharmacologically inactive, per se, is created by appropriate chemical modification of a parent drug, and it is chemically and/or biologically converted to the active parent drug after achieving its purpose [7,8]. This chemical approach has been widely utilized to improve the BA of hydrophobic drugs and/or the sustainability of drug action [7,8]. Prodrugs for the pharmaceutical purposes are likely to have PK profiles with improved linearity, low peak blood concentration, and sustained concentration of the parent drugs in the blood for a long time, resulting in extended duration of action and reduced risk of peak blood concentration-associated toxicity [7,8]. Besides, it would be ideal that prodrugs administered orally achieve the goal with minimal systemic absorption, thus avoiding possible side effects associated with systemic absorption of prodrugs [7,8].

To address the challenges associated with the PK of celecoxib, we designed hydrophilic prodrugs of celecoxib, which can release the parent drug in a controlled manner in the small and large intestine [9,10,11,12]. Hydrophilicity and the controlled release property of prodrugs are expected to not only improve the dissolution of the BCS class II drug but also sustain the absorption of the parent drug while limiting the systemic absorption of the prodrug in the intestine. Our previous studies have demonstrated that the amide bond formed between the carboxylic group of amino acids and the sulfonamide group of celecoxib is susceptible to the host and/or microbial enzymes in the intestine [10,11,12,13,14,15]. Therefore, celecoxib was coupled with hydrophilic amino acids to yield aspart-1yl celecoxib (A1C), glutam-1yl celecoxib (G1C), and *N*-glycyl-aspart-1yl celecoxib (N-GA1C). The ability of the hydrophilic celecoxib derivatives to improve the PK properties of the parent drug was evaluated. Moreover, we examined if such improvement in PK properties translates into an improved therapeutic action of celecoxib against carrageenan-induced paw edema in rats.

## 2. Materials and Methods

### 2.1. Synthesis of Aspart-1-yl Celecoxib (A1C) and Glutam-1-yl Celecoxib (G1C)

Briefly, 1,1′-carbonyldiimidazole (5.80 mmol) (CDI, Sigma-Aldrich, St. Louis, MO, USA) was added to 30 mL of acetonitrile (ACN, Junsei, Tokyo, Japan) containing 4.98 mmol of 4-benzyl *N*-(t-Boc)-aspartic acid or 5-benzyl *N*-(t-Boc)-glutamic acid (Tokyo Kasei Kogyo Co. Tokyo, Japan). After stirring for 10 min at room temperature (RT), 1.31 mmol of celecoxib (ether-extracted from Celebrex capsules) and 0.3 mL of triethylamine (TEA, Junsei) were added to the mixture, and the reaction was allowed to proceed while stirring for 4 h at 55 °C under nitrogen gas. After evaporating the solvents, the residue was dissolved in 40 mL ethyl acetate/ether (1:3), washed with 5% NaHCO_3_, and subsequently dried over anhydrous Na_2_SO_4_ followed by the evaporation of the solvents. For the deprotection of the t-Boc group, 1 M HCl/acetic acid (10 mg/mL) was treated for 3 h at RT, and for the deprotection of the benzyl group, 1 M NaOH (5 mL) was treated for 2 h at RT. After acidification, A1C or G1C was acquired as a white precipitate. 1H NMR spectra were obtained on a Varian AS 500 spectrometer (Varian, Palo Alto, CA, USA) and chemical shifts represent ppm downfield from tetramethylsilane. Infrared (IR) spectra were recorded on a Varian Fourier-transform IR spectrophotometer (Varian) and elemental analysis was performed using FLASH 2000 series (Thermo Scientific Inc. Waltham, MA). Physical data, including the melting point and the results of IR (Nujol), 1H-NMR (DMSO-*d*6), and elemental analysis, are identical with those in a previous study [9].

### 2.2. Synthesis of N-Glycyl-Aspart-1-yl Celecoxib (N-GA1C)

CDI (2.21 mmol) was dissolved in 12 mL of ACN containing 1.9 mmol of *N*-(t-Boc)-glycine (Tokyo Kasei Kogyo Co.). After stirring for 10 min at RT, 0.5 mmol of 4-benzyl aspart-1-yl celecoxib and 1.64 mL of TEA were added to the reaction mixture, and the mixture was stirred for 4 h at 55 °C. After the removal of solvents by evaporation, the residue was dissolved in 20 mL of ethyl acetate/ether (1:3), washed with 5% NaHCO_3_, dried over anhydrous Na_2_SO_4_, and then concentrated by evaporation. The residue was deprotected in 1 M HCl/acetic acid (10 mg/mL) at RT for 3 h and in 1 M NaOH (5 mL) for 2 h. After acidification, *N*-glycyl-aspart-1-yl celecoxib (N-GA1C) was acquired as white precipitate. Physical data, including the melting point and the results of IR (Nujol), 1H-NMR (DMSO-*d6*), and elemental analysis, are identical with those in a previous study [10].

### 2.3. HPLC Analysis

The HPLC system consisted of a model 306 pump, a 117 variable ultraviolet detector, a model 234 autoinjector, and a Model 805 manometric module from Gilson Inc. (Middleton, WI, USA). A C18 symmetry column (Waters, Milford, MA, USA) (250 × 4.6 mm) with a guard column (Waters, 3.9 × 20 mm) was used. The samples were filtered through a membrane filter (0.45 μm), and the filtrate (20 μL) was injected on a symmetry C18 column (Waters), which was eluted with a mobile phase at a flow rate of 1 mL/min. The mobile phase consisted of 60% ACN (Merck, Darmstadt, German) in 0.067 M phosphate buffer (pH 4.5) containing 0.1% trifluoroacetic acid (Sigma-Aldrich), which was filtered through a 0.45 μm membrane filter (Waters) before use. The eluate was monitored at 273 nm, and the detection limit was about 0.2 μg/mL under our experimental conditions. Accuracy and relative standard deviations were 98.7% and 0.43%, respectively Trilution^®^ LC V4 software (Gilson, Middleton, WI, USA) was used for data analysis. The retention times of celecoxib, A1C, G1C, and N-GA1C were 10.68, 3.11, 3.00, and 2.43 min, respectively.

### 2.4. Apparent Distribution Coefficient (Logd_6.8_) Measurement

For this study, 1-octanol and pH 6.8 isotonic phosphate buffer (IPB) were pre-saturated with IPB and 1-octanol, respectively. 1-octanol (10 mL) was added to 500 μM of celecoxib, A1C, or N-GA1C in 10 mL IPB. The mixture was shaken for 1 h and left at 37 °C for 3 h. The concentration of celecoxib, A1C, G1C, or N-GA1C in the aqueous phase was analyzed by HPLC. The apparent partition coefficients were calculated using the equation (Co − Cw)/Cw, where Co and Cw represent the initial and equilibrium concentration of the drug in the aqueous phase, respectively.

### 2.5. Chemical Stability Study

A1C, G1C, or N-GA1C (500 μM) in hydrochloric acid (pH 1.2) or isotonic phosphate (pH 6.8) buffer was incubated at 37 °C for 10 h. At the predetermined time points, 20 μL of the solution was transferred into an HPLC vial, and the concentrations of A1C, G1C, or N-GA1C were analyzed by HPLC.

### 2.6. Animals

The study animals were cared for following national and local guidelines. The animal protocol used in this study was reviewed and approved by the Pusan National University Institutional Animal Care and Use Committee on ethical procedures and scientific care (Approval number: PNU-2017-1525). All animals were obtained from Samtako (Osan, South Korea) and housed under pathogen-free conditions in the animal facility at the College of Pharmacy of Pusan National University. Rats were assigned randomly to the experimental groups. The investigators were not blinded to allocation during experiments and outcome assessment unless the section mainly included a blind assessment.

### 2.7. Drug Release Study

Drug release study was performed with the celecoxib derivatives as described previously [9,10]. Six-week-old male Sprague Dawley rats (250–260 g) were sacrificed using CO_2_, and a midline incision was made. The contents of the proximal small intestine, distal small intestine, and cecum, liver homogenate, or blood plasma were collected and suspended in IPB (20% (*w*/*v*)). A1C, G1C, or N-GA1C in the buffer (0.5 mL, 1 mM) was mixed with 0.5 mL of the suspension and then incubated at 37 °C under nitrogen gas. At the predetermined time points, the samples were extracted with 0.5 mL of ethyl acetate followed by centrifugation at 6000× *g* for 5 min. Methanol (1.0 mL) was added to the residue obtained from the evaporation of 0.1 mL of the organic layer. The solution was centrifuged at 20,000× *g* at 4 °C for 10 min. The concentrations of celecoxib, A1C, G1C, and N-GA1C in 20 μL of the supernatant were determined by HPLC.

### 2.8. Pharmacokinetic Study

Six-week-old male Sprague Dawley rats (250–260 g) were administered 10 mg/kg of celecoxib, A1C (equivalent mass of celecoxib), G1C (equivalent mass of celecoxib), or N-GA1C (equivalent mass of celecoxib) orally or intravenously via the tail vein. Blood samples were collected from the jugular vein using a heparinized syringe at predetermined times. Heparinized blood samples were immediately centrifuged at 6000× *g* for 5 min. Five-fold volume of ethyl acetate was mixed with plasma, centrifugation was performed at 6000× *g* for 5 min, and 1 mL of the organic solvent was evaporated. The residues were mixed with 0.1 mL of IPB and centrifuged at 20,000× *g* for 10 min at 4 °C. The concentration of celecoxib or N-GA1C in the samples was determined by HPLC. PK profiling was performed using PKsolver [16]. Absolute BA and relative BA were calculated using the following equations: Absolute BA (F, %) = ((AUC_0–∞_)PO/dose PO)/((AUC_0–∞_)IV/dose IV); Relative BA (%) = ((AUC_0–∞_)A/dose A)/((AUC_0–∞_)B/dose B).

### 2.9. Carrageenan-Induced Inflammation Study

Six-week-old male Sprague Dawley rats (250–260 g) were orally administered celecoxib (10 mg/kg), A1C (equivalent mass of celecoxib), G1C (equivalent mass of celecoxib), N-GA1C (equivalent mass of celecoxib), or IPB as a control. After 1 h, 0.1 mL of carrageenan (1%) was injected into the hind paw of the rats. Carrageenan-induced increase in paw volume (paw edema) was measured at predetermined time points (0, 3, 6, 12, and 24 h). The paw volume of rats up to the knee joint was measured using a plethysmometer. The degree of inhibition of paw edema (%) at each time point was calculated using the following equation: (Control volume − treatment group volume)/Control paw volume × 100.

### 2.10. Statistical Analysis

All experiments were performed at least twice with duplicate repeated measures. The results are expressed as means ± standard error of means. A one-way or two-way ANOVA, followed by Tukey’s HSD multiple comparison post hoc test was used to determine the differences among groups. Data were approximately normally distributed, and the variance was similar between the groups. Statistical significance is indicated as * *p* < 0.05, ** *p* < 0.01, *** *p* < 0.001, and **** *p* < 0.0001. GraphPad Prism 8.0 (GraphPad Software, La Jolla, CA, USA) was used for statistical analyses.

## 3. Results

### 3.1. Preparation of Celecoxib Derivatives Coupled with Amino Acids

**Scheme 1:**
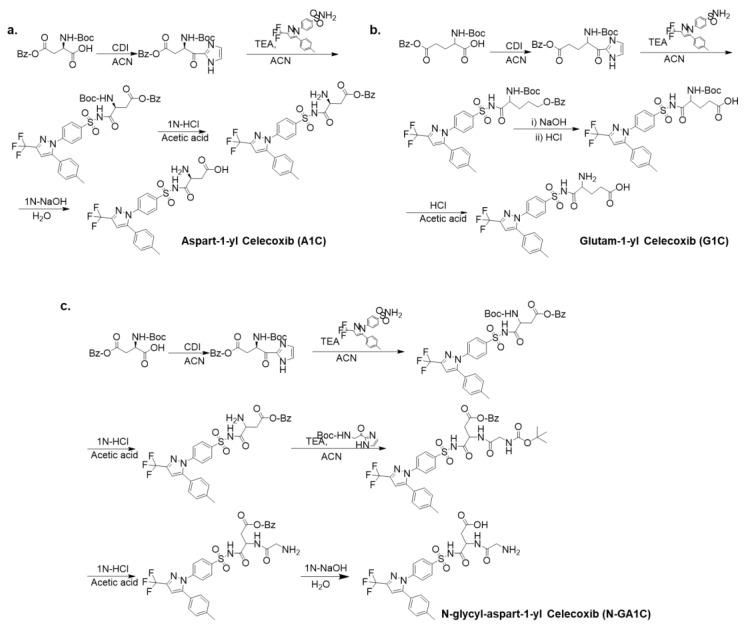
Synthesis scheme of aspart-1-yl celecoxib (A1C, **a**), glutam-1-yl celecoxib, (G1C, **b**), and *N*-glycyl-aspart-1-yl celecoxib (N-GA1C, **c**). Adapted from [9,10], published by Springer Nature, 2012 and Dovepress, 2015.

The synthesis of A1C, G1C (Scheme 1a) and N-GA1C (Scheme 1b) was conducted as described previously [9,10].We conjugated the sulfonamide group of celecoxib with the carboxyl group of protected aspartic acid and glutamic acid using CDI, a coupling reagent. The protection groups, t-Boc and benzyl groups, were removed in acidic and basic conditions, respectively, yielding A1C and G1C. For the synthesis of N-GA1C (Scheme 1b), the amine group of 4-benzyl aspart-1-yl celecoxib was conjugated with the carboxyl group of *N*-t-Boc glycine with CDI as the coupling reagent. Subsequently, the protection groups were removed, yielding N-GA1C. Physical data of the celecoxib-amino acid conjugates are shown in Table 1, which are identical with those in previous studies [9,10].

### 3.2. Celecoxib Derivatives Are Converted to Celecoxib in The Intestinal Contents of Rats

**Figure 1:**
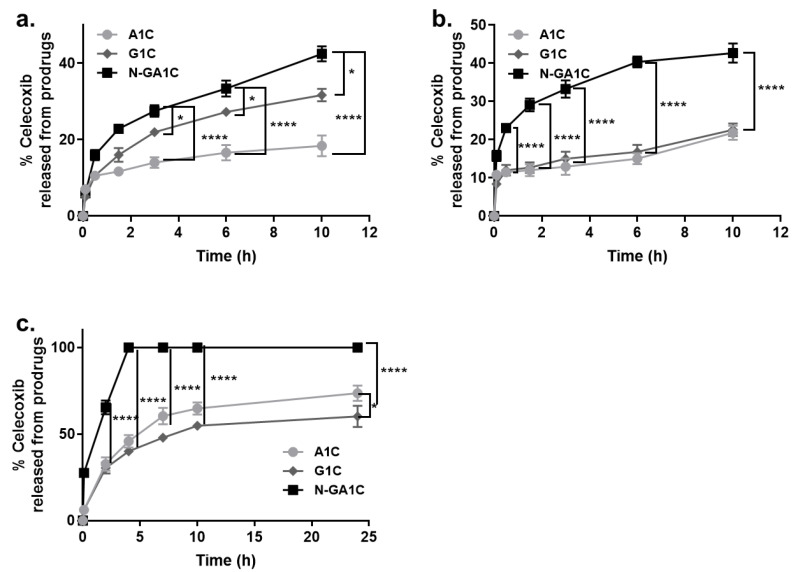
Amino acid conjugates of celecoxib release celecoxib in both small- and large-intestinal contents. (**a**–**c**) Percentage of celecoxib released from prodrugs after incubation with 20% suspension of contents of the proximal small intestine (**a**), distal small intestine (**b**), or cecal contents (**c**) in phosphate buffered saline (pH 6.8). Data are presented as mean ± s.e.m. from a representative experiment (*n* = 5) from 3 independent experiments. * *p* < 0.05, **** *p* < 0.0001, analyzed by two-way ANOVA with Tukey’s HSD multiple comparison post hoc test.

To examine if celecoxib derivatives can be converted to celecoxib in the intestine, A1C, G1C, and N-GA1C were incubated in the small- and large-intestinal contents and changes in the concentration of the celecoxib derivatives and celecoxib were monitored. In the proximal and distal small intestinal contents, N-GA1C released celecoxib (27.5 ± 1.51% at 3 h, PSI; 33.28% ± 2.25% at 3 h, DSI); the celecoxib release occurred at different rates -N-GA1C > G1C > A1C (p < 0.0001; Figure 1a,b). Upon incubation with cecal contents, N-GA1C was rapidly and entirely converted to celecoxib within 4 h, and the celecoxib release from N-GA1C was greater than from A1C and G1C that were partially converted to celecoxib even after 24 h (*p* < 0.0001; Figure 1c). These results are consistent with previous papers [9,10]. To rule out the possibility of chemical cleavage of the amide bonds between celecoxib and the amino acids, the celecoxib derivatives were incubated in HCl-NaCl buffer (pH 1.2) and IPB for 24 h before analyzing the concentration of celecoxib and the cececoxib derivatives. No change in concentration of the compounds was observed, suggesting that the celecoxib derivatives were chemically stable during GI transit. These results indicate that the release of celecoxib from A1C, G1C, and N-GA1C is mainly facilitated by intestinal enzymes in the small intestine and microbial enzymes in the large intestine.

### 3.3. Systemic Absorption and Exposure of Orally Administered N-GA1C Are Limited

**Figure 2:**
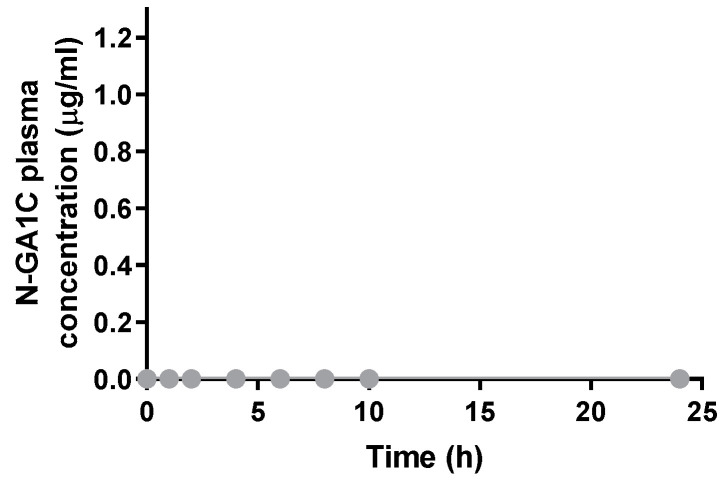
N-GA1C’s systemic absorption is limited after oral administration. Plasma concentration of N-GA1C was monitored after oral administration of N-GA1C (10 mg/kg). Data are presented as mean ± s.e.m. from a representative experiment (*n* = 5) from 2 independent experiments.

To examine if the systemic absorption of the hydrophilic celecoxib derivatives could be reduced, we measured the apparent distribution coefficients of A1C, G1C, N-GA1C, and celecoxib. The apparent distribution coefficients (logD_6.8_) were 0.6 (A1C), 0.8 (G1C), –0.13 (N-GA1C), and 4.01 (celecoxib), suggesting that the passive transport of the celecoxib derivatives via the intestinal epithelial layer is less efficient than that of celecoxib. N-GA1C was found to be the most effective derivative with regard to limiting the systemic absorption of celecoxib. To further prove whether N-GA1C’s systemic absorption is limited, we monitored blood levels of N-GA1C after oral N-GA1C. Figure 2 shows that N-GA1C was not detected in the plasma for 24 h, indicative of limited systemic absorption and exposure of N-GA1C. (Figure 2).

### 3.4. Celecoxib Derivatives are Metabolized to Celecoxib in the Liver Homogenate of Rats

**Figure 3:**
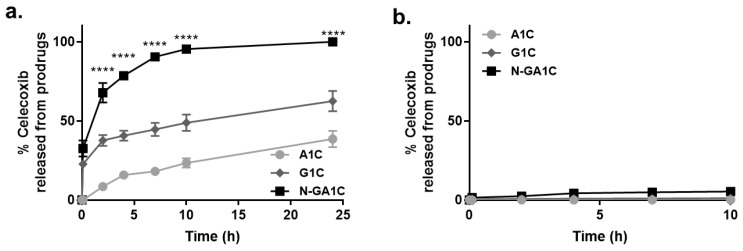
Celecoxib release profiles of A1C, G1C, and N-GA1C in liver homogenate and plasma. (**a**,**b**) Percentages of celecoxib released from the prodrugs after incubation with 20% suspension of liver homogenate (**a**) or blood plasma (**b**) in IPB. Data are presented as mean ± s.e.m. from a representative experiment (*n* = 5) from 2 independent experiments. **** *p* < 0.0001, analyzed by two-way ANOVA with Tukey’s HSD multiple comparison post hoc test.

Additionally, we examined if the celecoxib derivatives could be metabolized to celecoxib in the blood and liver if absorbed systemically. To do this, the celecoxib derivatives were incubated in the liver homogenate of rats, and the concentration of celecoxib released from the derivatives was analyzed. N-GA1C was metabolized to celecoxib (94.87 ± 1.78% after 10 h and 100% conversion at 24 h; Figure 3a), indicating that it was more susceptible to hepatic metabolism than A1C and G1C (Figure 3a). On incubation with plasma for 24 h, none of the derivatives released celecoxib (Figure 3b). These results indicate that N-GA1C was poorly absorbed and a large portion of N-GA1C absorbed systemically would be rapidly converted to celecoxib in the liver.

### 3.5. Celecoxib Derivatives Improve the PK Properties of Celecoxib

**Figure 4:**
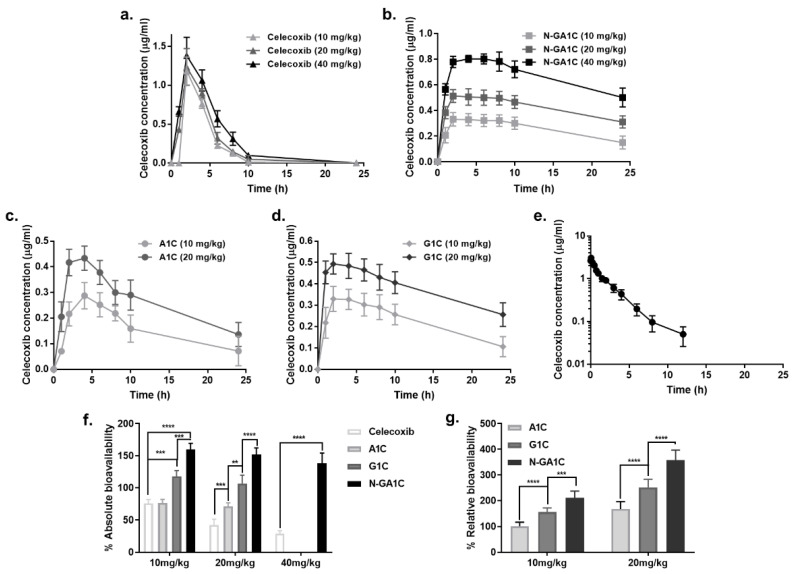
Celecoxib derivatives improve the PK properties of celecoxib. (**a**–**e**), Plasma concentration of celecoxib after the oral administration of celecoxib (**a**), N-GA1C (**b**), A1C (**c**), or G1C (**d**). (**e**), Plasma concentration of celecoxib after the intravenous administration of celecoxib. (**f**–**g**), Absolute bioavailability (**f**) and relative bioavailability (**g**) of A1C, G1C, and N-GA1C after the oral administration of celecoxib, A1C (equivalent mass of celecoxib), G1C (equivalent mass of celecoxib), or N-GA1C (equivalent mass of celecoxib) to Sprague-Dawley rats. Data are presented as mean ± s.e.m. from a representative experiment (*n* = 5) from 3 independent experiments. ** *p* < 0.01, *** *p* < 0.001, and **** *p* < 0.0001, analyzed by two-way (**a**–**d**) or one-way (**e**–**f**) ANOVA with Tukey’s HSD multiple comparison post hoc test.

Our data suggest that N-GA1C would release celecoxib during transit in the small and large intestine with the least systemic absorption. Next, we examined if N-GA1C improved the PK properties of celecoxib. First, the blood concentration of celecoxib was monitored after the oral administration of celecoxib and N-GA1C to rats. To see how the differences in the release rate of celecoxib in the intestinal contents and the distribution coefficient (indicating aqueous solubility) affect the PK properties, the same experiment was performed with A1C and G1C. After the oral administration of N-GA1C at 10, 20, and 40 mg/kg, sustained profiles of celecoxib concentration were observed in the blood, and the peak concentration of celecoxib (Cmax) was reduced from 1.18 μg/mL (with oral celecoxib at 10 mg/kg) to 0.33 μg/mL (with oral N-GA1C at 10 mg/kg) (Figure 4a,b, Table 2). The blood concentration of celecoxib after the oral administration of N-GA1C was higher than that observed after the oral administration of celecoxib from 6 h until the end of the experiment; the blood concentration of celecoxib rapidly decreased after the oral administration of celecoxib. Although the blood concentration of celecoxib was sustained for 24 h with both A1C and G1C, N-GA1C afforded greater concentration of celecoxib (0.15–0.33 μg/mL) than the other celecoxib derivatives (A1C, 0.08–0.29 μg/mL and G1C, 0.10–0.31 μg/mL) (Figure 4c,d), suggesting that the more hydrophilic and faster the bioconversion of a celecoxib-amino acid conjugate is, the higher the ability of the conjugate to sustain a high blood concentration of celecoxib.

Next, we examined if the sustained blood concentration of celecoxib after the oral administration of N-GA1C is linked to improving oral BA of celecoxib. To calculate the relative and absolute BA of N-GA1C, area under the curve (AUC) values were acquired in advance after the intravenous and oral administration of various doses of celecoxib (Figure 4a,e, and Table 2). For comparison with the other celecoxib derivatives, the relative and absolute BA of A1C and G1C was calculated at 10 and 20 mg/kg.

As the oral doses of celecoxib increased from 10 to 40 mg/kg, the absolute BA of celecoxib dramatically decreased from 75.82 ± 2.66% to 29.08 ± 2.02% (Figure 4a,e,f, and Table 2). However, oral N-GA1C achieved greater absolute BA of celecoxib than oral celecoxib at all doses (*p* < 0.0001), and no significant differences in absolute BA were observed among the different doses of N-GA1C (Figure 4a,b,e,f and Table 2). A1C and G1C at 20 mg/kg increased the absolute BA of celecoxib by up to approximately two- (for A1C) and three-fold (for G1C), whereas no increase or a 30% increase in absolute BA was observed with oral A1C and oral G1C at 10 mg/kg, respectively, which was significantly lesser than that of N-GA1C at the same dose (Figure 4a,c–f and Table 2). Moreover, the relative BA of oral N-GA1C was determined to evaluate the improvement in the linearity of PK profiles of celecoxib. With oral N-GA1C, the relative BA of celecoxib increased from 210.76 ± 12.07% to 476.76 ± 12.07% (Figure 4a,b,f and Table 2) with the elevation of dose from 10 mg/kg to 40 mg/kg. Increased relative BA was also observed with A1C and G1C as the doses were elevated from 10 mg/kg to 20 mg/kg, and their dose proportionality was similar to that of N-GA1C (Figure 4g). Overall, these results suggest that the celecoxib derivatives can improve the PK properties of celecoxib, and N-GA1C is the most promising prodrug of celecoxib to overcome the challenges associated with the PK of celecoxib.

### 3.6. Oral N-GA1C Prolongs and Enhances the Anti-Inflammatory Activity of Celecoxib

**Figure 5:**
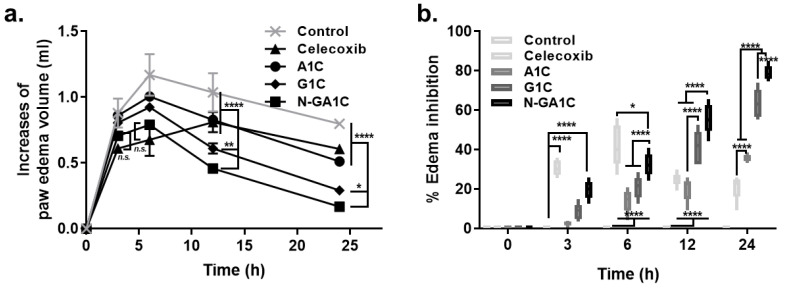
N-GA1C prolongs and enhances the anti-inflammatory activity of celecoxib. (**a,b**), SD rats were orally administered celecoxib (10 mg/kg), A1C (equivalent mass of celecoxib), G1C (equivalent mass of celecoxib), N-GA1C (equivalent mass of celecoxib), or IPB. (**a**), Changes in rat paw volume in each group for 24 h. (**b**), Percentage changes in edema inhibition in each group for 24 h. Data are presented as mean ± s.e.m. from a representative experiment (*n* = 5) from 2 independent experiments. * *p* < 0.05, ** *p* < 0.01, and **** *p* < 0.0001, analyzed by two-way ANOVA with Tukey’s HSD multiple comparison post hoc test.

Although oral N-GA1C significantly improved the PK properties of oral celecoxib, it is not certain that such improvement confers therapeutic benefits on celecoxib. Thus, we compared the therapeutic (anti-inflammatory) activity of oral N-GA1C with that of oral celecoxib in a carrageenan-induced rat paw edema model [17,18]. Except for the linearity of the PK profiles, N-GA1C exhibited greater absolute BA and higher sustained concentration of celecoxib in the blood than the other celecoxib derivatives. To determine the relevance of PK data to therapeutic activity, the same experiment was performed with G1C and A1C. The anti-inflammatory activity was determined by calculating volume reduction of rat paw edema after the oral administration of each drug (10 mg/kg). With oral celecoxib, the volume reduction of paw edema reached the maximum around 6 h (42.06 ± 4.69%) and continuously diminished to 20.99 ± 2.791% after 24 h (Figure 5). Oral N-GA1C achieved maximal volume reduction of paw edema (78.96 ± 1.56%) at 24 h, which was greater than that achieved by oral celecoxib. However, the volume reduction achieved by N-GA1C at 6 h (32.42 ± 2.50%) was less than that by celecoxib (Figure 5). Oral G1C and A1C showed a similar pattern to oral N-GA1C in terms of their ability to reduce the volume of rat paw edema. G1C reduced the edema volume to a greater extent than oral celecoxib from 12 h, whereas A1C could only achieve this at 24 h. The other celecoxib derivatives were less effective in reducing the volume of rat paw edema than N-GA1C at all time points. Compared with celecoxib and the other celecoxib derivatives, oral N-GA1C showed greater anti-inflammatory activity (Figure 5). Moreover, the anti-inflammatory activities of the celecoxib derivatives correlated with their PK properties such as BA and sustained the concentration of celecoxib in the blood.

## 4. Discussion

Celecoxib, sold under the brand name Celebrex, is a COX-2 selective nonsteroidal anti-inflammatory drug widely used for the treatment of pain and inflammation in chronic inflammatory diseases such as osteoarthritis, rheumatoid arthritis, and ankylosing spondylitis [1,2]. Despite its extensive clinical use, there are some challenges associated with the PK of celecoxib, such as low BA, poor linearity in PK profiles, and peak blood concentration-related cardiovascular toxicity, thereby deterring broader clinical usage [2,3,4,5,6]. To address these issues, we designed celecoxib prodrugs, namely A1C, G1C, and N-GA1C, with high hydrophilicity and the ability to convert to celecoxib in the intestine (Scheme 1).

Consistent with our previous studies, the conjugation of celecoxib with amino acids via the amide bond of the sulfonamide group in celecoxib and the carboxyl group in amino acids yielded prodrugs bio-activated in the small and large intestine [10,11,12,13,14,15]. This is shown in our data demonstrating that A1C, G1C, and N-GA1C liberated celecoxib in the small and large intestinal contents upon incubation. The derivatives were stable in buffer solutions of pH 1.2 and 6.8, indicating chemical stability and enzyme susceptibility of the amide bonds. The enzymatic susceptibility of the prodrugs was dependent on the amino acid promoieties as intestinal bioconversion of the celecoxib derivatives occurred at different rates (N-GA1C > G1C > A1C). This result also indicates that the dipeptide promoiety (*N*-glycyl-aspart-1-yl) in N-GA1C is not bio-activated to celecoxib by one-by-one cleavage to form A1C as the intermediate. Actually, the presence of A1C was not observed during the incubation of N-GA1C in the intestinal contents.

The cleavage of the celecoxib derivatives to celecoxib in the small- and large-intestinal contents and the hydrophilicity of the celecoxib derivatives, indicated by their lower distribution coefficient than that of celecoxib, suggest that oral celecoxib derivatives constantly release celecoxib during transit in the small and large intestine. The release of celecoxib occurs with limited systemic absorption of the celecoxib derivatives, especially for N-GA1C, which has the lowest DC. Accordingly, compared with oral celecoxib, the oral celecoxib derivatives could more effectively sustain the concentration of celecoxib in the blood for 24 h. Furthermore, the concentration of celecoxib after the oral administration of N-GA1C was higher than that measured after the oral administration of G1C and A1C at all time points. This is consistent with the release profiles of celecoxib from the celecoxib derivatives in the intestinal contents, where N-GA1C released celecoxib faster than the other derivatives. The data showing that oral G1C achieved greater concentration of celecoxib in the blood than oral A1C further supports our findings.

The celecoxib derivatives increased the BA of celecoxib as determined by AUC. Our data show that the BA of the derivatives was achieved in the following order, N-GA1C > G1C > A1C, suggesting that the BA of celecoxib depends on both the hydrophilicity and the rate of celecoxib release in the intestine. It is likely that N-GA1C provides higher concentration of celecoxib available for systemic absorption than the other derivatives; this is due to greater hydrophilicity, resulting in better dissolution and faster release of celecoxib. At the same time, sustained release of celecoxib from N-GA1C in the intestine may reduce the precipitation of celecoxib due to low solubility. This argument is reasonable considering that celecoxib is a BCS class II drug, with low solubility and high permeability; thus, an increase in the amount of celecoxib available for absorption increases the systemic absorption. Furthermore, it is not surprising that the BA of celecoxib was greater with oral G1C than with A1C, given that the bioconversion of G1C (to celecoxib) occurred faster than that of A1C and DCs of the two derivatives were similar.

The systemic absorption of N-GA1C is lower than that of the other celecoxib derivatives owing to its lowest DC; hence, contribution of hepatic N-GA1C (absorbed from intestine) metabolism toward BA increase would be negligible, unlike G1C and A1C whose systemic absorption may be more than N-GA1C. In addition, among the celecoxib derivatives, the most hydrophilic N-GA1C has the least risk of systemic side effects. Our data showed that N-GA1C is the most susceptible to hepatic metabolism, thus further reducing the risk of systemic side effects of the prodrug [7,8].

Our data showing that an increase in the dose of N-GA1C from 10 mg/kg to 40 mg/kg was accompanied by proportional increase in PK parameters such as Cmax and AUC indicate that N-GA1C improves the BA as well as the linearity of the PK profile of celecoxib. This suggests that the pharmaceutical behavior of N-GA1C in the intestine is not significantly changed in the dose range. Considering that a linear PK profile facilitates the prediction of concentration-time profiles and thus enables the adjustment of dose and dose regimen in patients, N-GA1C may be more suitable for therapeutic use than celecoxib, especially for patients who need dose adjustment for celecoxib. Although A1C and G1C also showed similar proportionality with regard to dose change, their PK values were less than those of N-GA1C. The difference in PK improvement shown by the celecoxib derivatives is associated with their anti-inflammatory activities.

The improvement in the PK properties of celecoxib positively affects its therapeutic activity and duration of action. This is supported by the data demonstrating that, compared with oral celecoxib, oral N-GA1C exhibited greater anti-inflammatory activity for a longer duration in the carrageenan-induced rat paw edema model, which was consistent with the concentration profile of celecoxib in the blood after the oral administration of N-GA1C and celecoxib. Furthermore, of all the derivatives, N-GA1C exhibited the highest ability to reduce rat paw edema at all time points, ensuring close association of PK improvement with therapeutic activity and duration.

Taken together, the celecoxib derivatives, N-GA1C, G1C, and A1C, are prodrugs of celecoxib with improved PK properties that can be largely ascribed to the continuous bioconversion of the celecoxib derivatives to celecoxib during transit through the intestine, thereby leading to the sustained absorption of celecoxib. Compared with celecoxib and the other derivatives, N-GA1C exhibited superior anti-inflammatory activity and lower risks of side effects due to limited systemic absorption; hence, it is the most promising prodrug of celecoxib. The prodrug strategy may be an effective approach to improve the PK and therapeutic properties of other BCS class II hydrophobic drugs with pharmaceutical limitations similar to those of celecoxib.

## Figures and Tables

**Table 1:** IR spectra, NMR spectra, elemental analysis, log D_6.8_ values and melting point (MP) of celecoxib, A1C, G1C, and N-GA1C.

	IR Spetra (Nujol) (cm^−1^)	^1^H-NMR (DMSO-*d*6) (δ, ppm)	Elemental Analysis	Log D_6.8_	Melting Point
Celecoxib	1720 (C=O, SO_2_NHCO)	2.30 (s, 3H), 7.17 (s, 1H), 7.16–7.20 (m, 4H), 7.51 (d, 2H, J = 7.5 Hz), 7.86 (d, 2H, J = 8.0 Hz)	C_19_H_17_F_3_N_4_O_3_S (438); calcd: C, 52.05; H, 3.88; N, 12.78; found: C, 52.10; H, 3.89; N, 12.75.	4.01	189 °C–194 °C
A1C	1721 (C=O, SO_2_NHCO), 1596 (C=O, zwitterionic carboxylate salt)	1.98 (dd, 1H, J = 10.5, 16.5 Hz), 2.29 (s, 3H), 2.32 (dd, 1H, J = 3.5, 16.0 Hz), 3.34 (m, 1H), 7.13 (s, 1H), 7.15–7.20 (m, 4H), 7.34 (d, 2H, J = 8.5 Hz), 7.80 (d, 2H, J = 9.0 Hz)	C_21_H_19_F_3_N_4_O_5_S (496.10) Calcd: C, 50.80; H, 3.86; N, 11.29; Found: C, 50.82; H, 3.91; N, 11.35.	0.6	134 °C–137 °C
G1C	1719 (C=O, SO_2_NHCO), 1595 (C=O, zwitterionic carboxylate salt)	1.7 (m, 1H), 2.25 (m, 1H), 2.02 (m, 2H), 2.30 (s, 3H), 3.29 (m, 1H), 7.14 (s, 1H), 7.16–7.21 (m, 4H), 7.59 (d, 2H, J = 8.5 Hz), 7.95 (d, 2H, J = 8.5 Hz)	C_22_H_21_F_3_N_4_O_5_S (510.12) Calcd: C, 51.76; H, 4.15; N, 10.98; Found: C, 51.82; H, 4.11; N, 11.20.	0.81	110 °C–115 °C
N-GA1C	1700 (C=O, SO_2_NHCO), 1668 (C=O, amide), 1596 (C=O, carboxylic)	2.30 (s, 3H), 2.35 (dd, 1H, J = 7.5, 15.5 Hz), 2.61 (dd, 1H, J = 5.5, 12.5 Hz), 4.02 (t, 2H, J = 5.0 Hz), 4.4 (m, 1H), 7.13 (s, 1H), 7.14–7.21 (m, 4H), 7.51 (d, 2H, J = 8.5 Hz), 7.85 (d, 2H, J = 8.0 Hz)	C_23_H_22_F_3_N_5_O_5_S (553.12) Calcd: C, 49.91; H, 4.01; N, 12.56; Found: C, 49.95; H, 4.07; N, 12.50.	−0.13	154 °C–158 °C

**Table 2:** Pharmacokinetic profiles of celecoxib, A1C, G1C, and N-GA1C. Absolute bioavailability and relative bioavailability were calculated using the following equations: Absolute bioavailability (F, %) = [(AUC_0–∞_)PO/dose PO]/[(AUC_0–∞_)IV/dose IV]; Relative bioavailability (%) = [(AUC0–∞)A/dose A]/[(AUC_0–∞_)B/dose B].

	Dose (mg/kg)	Cmax (mg/mL)	Tmax (h)	AUC_0–24_ (mg/mL)h	AUC_0–∞_ (mg/mL)h	CL (mL/min/kg)	Vd (L/kg)	BA % (f)	t1/2 (h)	Relative Bioavailability %
Celecoxib (IV)	10	-	-	5.99 ± 0.44	6.12 ± 0.52	8.01	1.04	-	1.50	
Celecoxib (Oral)	10	1.18 ± 0.18	2	4.60 ± 0.45	4.64 ± 0.45	-	-	75.82% ± 2.66		-
Celecoxib (Oral)	20	1.23 ± 0.24	2	5.06 ± 0.49	5.19 ± 0.50	-	-	42.40% ± 3.94		-
Celecoxib (Oral)	40	1.38 ± 0.24	2	6.72 ± 0.58	7.12 ± 0.69	-	-	29.08% ± 2.02		-
A1C (Oral)	10	0.29 ± 0.05	4	3.68 ± 0.39	4.68 ± 0.43	-	-	76.47% ± 2.48		100.85% ± 7.07
A1C (Oral)	20	0.43 ± 0.05	4	6.32 ± 0.53	8.70 ± 0.72	-	-	71.07% ± 2.51		167.62%± 12.84
G1C (Oral)	10	0.31 ± 0.06	2	5.35 ± 0.44	7.22 ± 0.61	-	-	117.97% ± 4.032		155.59% ± 7.16
G1C (Oral)	20	0.49 ± 0.05	2	9.06 ± 0.85	13.07 ± 1.11	-	-	106.78% ± 5.80		251.84%± 14.15
N-GA1C (Oral)	10	0.33 ± 0.05	2	6.09 ± 0.49	9.78 ± 0.84	-	-	159.80% ± 4.31		210.76% ± 12.07
N-GA1C (Oral)	20	0.51 ± 0.05	2	10.23 ± 0.94	18.55 ± 1.19	-	-	151.55% ± 4.75		357.43% ± 17.74
N-GA1C (Oral)	40	0.80 ± 0.04	4	15.75 ± 1.12	33.87 ± 3.69	-	-	138.35% ± 7.079		476.76% ± 21.15
